# Exploring Breast Cancer Survivors’ Preferences for Text Messaging–Based Mobile Health Interventions Targeting Sleep and Physical Activity: Qualitative Study

**DOI:** 10.2196/85386

**Published:** 2026-07-09

**Authors:** Chi-shan Tsai, Warren Szewczyk, HyunHae Lee, Michelle Drerup, Erin Abu-Rish Blakeney, Heather Greenlee, Jaimee L Heffner, Alexi Vasbinder, Kerryn W Reding

**Affiliations:** 1 School of Nursing, University of Washington, 1959 NE Pacific St, Box 357266, Seattle, WA, 98195, United States, 1 206-221-1571; 2 Neurological Institute, Cleveland Clinic, Cleveland, OH, United States; 3 Public Health Sciences Division, Fred Hutchinson Cancer Center, Seattle, WA, United States; 4 Clinical Research Division, Fred Hutchinson Cancer Center, Seattle, WA, United States; 5 School of Medicine, University of Washington, Seattle, WA, United States

**Keywords:** breast cancer, sleep disturbance, physical activity, technology preference, thematic analysis

## Abstract

**Background:**

Sleep disturbances and low physical activity are common among breast cancer (BC) survivors and are associated with increased morbidity and mortality. Given the increased access to technological devices and the growing popularity of SMS text messaging–based mobile health interventions, these tools have the potential to both address sleep disturbances and promote physical activity in a scalable and cost-effective way. To understand and make effective use of these tools, it is important to consider the preferences of BC survivors with sleep disturbances, including how SMS text messaging–based mobile health interventions could deliver interventions involving physical activity and sleep hygiene.

**Objective:**

The objective of this study was to explore the perspectives and preferences of BC survivors regarding text messaging–based mobile interventions targeting sleep and physical activity.

**Methods:**

Three focus groups (n=13 participants) and 3 individual interviews (n=3) were conducted from May 2020 to March 2021 with 16 BC survivors (mean age=59.3, SD 8.9 y) currently experiencing sleep disturbances. The interview questions focused on their experiences with poor sleep and preferences for text messaging–based mobile health interventions. Thematic analysis was applied to the deidentified transcriptions of audio recordings.

**Results:**

Three themes were identified: (1) attitudes toward health interventions delivered through text messaging, (2) specific user needs, and (3) technology usage habits and preferences. Most participants reported a positive attitude toward the possibility of using technology to help improve their sleep and increase their physical activity. Most expressed a high level of acceptance toward some technologies, such as text messaging and mobile apps, but not others, such as voice interactions. In terms of desired features, reminders and accountability features, such as meeting physical activity goals, were mentioned most frequently. In addition, incorporating bedtime and relaxation exercise reminders was thought to be helpful. Regarding time and frequency, a daily reminder scheduled for 1 hour before bedtime was found to be acceptable.

**Conclusions:**

The insights have been used to guide the development of a messaging-based mobile health intervention for improving sleep and physical activity in BC survivors. Future research will focus on delivering an intervention addressing these health behaviors and assessing its acceptability and effectiveness.

## Introduction

More than 4 million breast cancer (BC) survivors currently reside in the United States [1]. In recent years, BC survival rates have substantially improved due to advancements in early detection and treatment [2]. However, BC survivors experience a variety of challenges following treatment. Sleep disturbances, defined as subjective difficulty with initiating or maintaining sleep, are one of the common challenges, as more than half of BC survivors report experiencing sleep disturbances [3-6]. Compared with the healthy population, BC survivors experience poorer sleep quality [7]. Sleep disturbances following a BC diagnosis have been associated with mental health problems such as anxiety and depression [8-10]. In addition, poor sleep quality is recognized as being related to the poor quality of life of BC survivors [11,12]. Therefore, it is crucial to develop effective interventions that specifically address sleep disturbances in this population.

To effectively alleviate these sleep disturbances, several nonpharmacological interventions have been investigated in prior research, such as cognitive behavioral therapy for insomnia (CBTi) and physical activity [13]. CBTi is a multicomponent intervention that is recommended as the first-line treatment for chronic insomnia disorder [14]. However, due to a shortage of skilled and trained CBTi providers, this treatment is underutilized [15]. Internet-based CBTi interventions have been developed and evaluated in several randomized controlled trials, including those targeting BC survivors, demonstrating encouraging results [16-18]. However, when comparing different CBTi formats, asynchronous or self-help interventions may still be less effective than synchronous CBTi formats (eg, face-to-face and telehealth) [19]. The challenges include high attrition and a lack of synchronous support due to the limited availability of trained CBTi providers [19]. In this context, technology-based tools may be able to reach a broader segment of patients with sleep problems and increase patient engagement by providing real-time interaction and personalized feedback, enabling more accessible dissemination of CBTi without relying on human resources.

In addition to CBTi, physical activity interventions such as yoga [20] or endurance training combined with resistance training [21] are common strategies for reducing sleep disturbances or improving sleep quality. Beyond improving sleep, studies have also linked physical activity to improved survival and quality of life in BC survivors [22]. A previous study reported that the majority of survivors were unable to maintain adequate levels of physical activity 10 years after diagnosis [22]. Hence, several studies have examined preferences toward technology-based interventions in this population to explore potential and effective strategies. For example, BC survivors reported high acceptability toward remotely delivered exercise programs and wearable activity trackers, valuing personalized feedback and self-monitoring features [23,24]. Although digital health interventions have shown potential for supporting health behavior change and improving health outcomes in populations without cancer, such as improvements in physical activity and mental health outcomes [25,26]. Messaging-based mobile health interventions represent a promising delivery approach. Some previous studies have demonstrated that delivering interventions via mobile phone messaging may improve physical activity among populations with cancer, including BC [27]. In addition, evidence from populations without cancer has supported their effectiveness in improving sleep outcomes [28,29]. However, further exploration is needed to understand how these interventions can be designed to address sleep disturbances and promote physical activity among BC survivors. Therefore, the aim of this study was to explore the perspectives and user preferences of BC survivors on the acceptability of using messaging-based mobile health interventions to address sleep disturbances, incorporating physical activity as one of the strategies.

## Methods

### Participants

Participants were recruited via Facebook advertisements and completed an online screening survey to assess eligibility. Following the completion of the online screening survey, research staff contacted potential participants by telephone. Inclusion criteria required that participants (1) were ≥18 years of age, (2) had a prior diagnosis of stage I-IV invasive BC, (3) self-reported poor sleep defined as a >4 global score on the Shortened Pittsburgh Sleep Quality Index [30], and (4) had an Eastern Cooperative Oncology Group Performance Status of 0 to 2 (indicating the ability to perform activities of daily living) [31]. The exclusion criteria were (1) a prior diagnosis of restless legs syndrome, periodic leg movement disorder, narcolepsy, rapid eye movement sleep behavior disorder, or sleep-related breathing disorder; or (2) active receipt of chemotherapy or radiation (endocrine therapy permitted). Sixty-four individuals filled out the initial screener. Of these, 22 participants provided consent, while 4 declined participation, 7 were found ineligible, and 31 were lost to follow-up due to being unreachable. Among those who consented, 2 participants withdrew after enrollment and 4 could not be scheduled for focus groups or interviews. Ultimately, a total of 16 BC survivors experiencing current sleep disturbances were included.

### Data Collection

Three online focus groups and 3 individual online interviews were conducted from May 2020 to March 2021. Two groups consisted of 4 participants each, while the third group comprised 5 participants. Individual interviews were conducted with 3 participants who had difficulty scheduling focus group times. All discussions were conducted and recorded via Zoom (Zoom Video Communications, Inc), and each discussion lasted approximately 60 minutes.

The focus groups were led by 2 authors (KWR and WS), and the individual interviews were conducted by WS. Group discussions were framed by semistructured subject guides (Table 1). The first section of each focus group and individual interview explored participants’ experiences with sleep disturbances and their coping strategies. Next, participants were asked about their preferences regarding potential features of sleep-related technology interventions, including the use of physical activity as one potential strategy to improve sleep.

Data saturation was assessed retrospectively following the completion of 3 focus groups and 3 individual interviews. The analysis showed consistent themes across sessions, with no new codes or conceptual categories identified in later transcripts. Consequently, no further data collection was required.

**Table 1.** Semistructured subject guides.

Topic	Questions
Current sleep information	Did you have sleep difficulties before breast cancer therapy or did they start with breast cancer therapy? What strategies have you used in the past to deal with insomnia? What are some things you wish you had known earlier when you first started having sleep difficulties? What barriers do you experience in getting help with your sleep?
Technology preferences	What do you like about texting? What don’t you like? Do you ever have any challenges with sending or writing text messages? How do you feel about receiving text messages related to your health? In general, how do you feel about text messaging? How do you feel about the following format and features? Which might you prefer? App on your phone that you proactively access to receive information Text message system that reaches out to you with information Going to a website and reading information Would you be willing to use an app or text–based intervention for 6 weeks? How comfortable are you discussing something personally difficult or distressing over text message? Would you feel comfortable bringing this up with a bot (software program)?
Data privacy concerns	Do you have any privacy concerns about receiving information via text message about your health? Do you have concerns about sharing health information over text? How do you feel about automatic data collection that happens in the “background”?
Specific feature questions related to the prototype of a chatbot	Would you be willing to answer questions each day about your sleep, if it will help the app make recommendations?

### Data Analysis

#### Statistical Analysis

The characteristics and medical histories of the participants were analyzed using descriptive analyses in Microsoft 365 Excel (Microsoft Corporation).

#### Qualitative Data Analysis

The audio recordings were transcribed verbatim and analyzed using thematic analysis, adhering to the 6-step process outlined by Braun and Clarke [32]. ATLAS.ti 23, a qualitative data analysis software developed by ATLAS.ti Scientific Software Development GmbH, was used to analyze qualitative data. The authors, CST and HL, familiarized themselves with the data through multiple readings of the group data. CST and HL conducted the thematic analysis on a subset of the transcripts to create an initial coding framework. Discrepancies were resolved through discussion and agreement or by consulting with KWR to finalize the themes and subthemes. Throughout the process, the themes were repeatedly reviewed and refined to ensure that they accurately reflected the data. Intercoder reliability was evaluated on a data subset, resulting in 93.2% intercoder agreement between coders (CST and HL), signifying a high level of consistency. Data from focus groups and individual interviews were analyzed separately. Focus groups were conducted to gather shared perspectives and encourage discussion among participants, while individual interviews explored more in-depth personal experiences. Themes were first developed from focus group data, then applied and refined against the individual interview data. The final themes provide a comprehensive understanding of the perspectives of BC survivors. The final set of themes and the report were then shared with other coauthors for feedback.

### Ethical Considerations

The study was reviewed and approved by the University of Washington Institutional Review Board (approval number 9642). All participants provided verbal informed consent, as written informed consent was waived. Since all the discussions were conducted and recorded via Zoom, participants were informed that sessions would be audio recorded. All transcripts were deidentified before data analysis. All participants received a $25 gift card as compensation.

## Results

### Overview

Three focus groups and 3 individual interviews were conducted with a total of 16 BC survivors experiencing sleep disturbances. The characteristics of the participants are summarized in Table 2. The participants were aged between 41 and 72 years; the average age was 59.3 (SD 8.9) years. Most of the participants were White (14/16, 87.5%), while the remainder were Black (2/16, 12.5%). Participants had varying levels of education, with 68.8% (11/16) holding a bachelor’s degree or higher; they also had varying stages of BC, with 50% (8/16) diagnosed with stage II BC. The majority had received chemotherapy, radiation, and/or hormone therapy.

**Table 2.** Participants’ demographic characteristics and medical history (N=16).

Characteristics	Values
Age (y), mean (SD)	59.3 (8.9)
Race, n (%)	
Black	2 (12.5)
White	14 (87.5)
Ethnicity, n (%)	
Not Hispanic or Latino	16 (100)
Hispanic or Latino	0 (0)
Education, n (%)	
GED^a^ or high school degree	1 (6.25)
Some college, no degree	3 (18.8)
Associate’s degree	1 (6.25)
Bachelor’s degree	8 (50)
Master’s degree	3 (18.8)
Work status, n (%)	
Unable to work	2 (12.5)
Retired	5 (31.2)
Working full time	8 (50.0)
Other	1 (6.25)
Marital status, n (%)	
Divorced	3 (18.8)
Domestic partnership	1 (6.2)
Married	10 (62.5)
Never married	2 (12.5)
Breast cancer stage at diagnosis, n (%)	
Stage 1	4 (25)
Stage 2	8 (50)
Stage 3	4 (25)
Years since diagnosis, mean (SD)	5.99 (5.83)
Received surgery related to breast cancer, n (%)	
Yes	16 (100)
No	0 (0)
Received radiation therapy, n (%)	
Yes	14 (87.5)
No	2 (12.6)
Received hormone therapy or antiestrogen therapy, n (%)	
Yes	11 (68.8)
No	5 (31.2)
Received Herceptin (HER2) treatment, n (%)	
Yes	2 (12.5)
No	14 (87.5)
History of breast cancer recurrence, n (%)	
No	11 (68.8)
Yes	5 (31.2)

a GED: General Educational Development.

Three themes emerged from the analysis: (1) attitudes toward the use of technology-based tools for promoting sleep and physical activity, (2) specific user needs for future SMS text messaging–based interventions, and (3) technology usage habits and preferences. Table 3 provides an outline of the themes, subthemes, and definitions of the themes.

**Table 3.** Outline of the themes, subthemes, and definitions of the themes.

Theme and subtheme	Definition
Theme 1: attitudes toward the use of technology-based tools for promoting sleep and physical activity	
1.1 Acceptance of new technology-based tools/strategies	The willingness of individuals to use new technology-based tools or strategies to improve sleep and physical activity
1.2 Concern about technology interfering with sleep	Individuals’ worries or hesitations regarding the utilization of sleep-related technology
1.3 Data privacy	Individuals’ perspectives on sharing personal sleep- and physical activity–related data
Theme 2: specific user needs for future SMS text messaging–based intervention	
2.1 Features	The functionalities and attributes that users look for in sleep- and physical activity–related technology
2.2 Time and frequency	Users’ preferences regarding when and how often they would engage with sleep- and physical activity–related technology
2.3 Motivation for engagement	The factors or functionalities that motivate users to regularly engage with sleep- and physical activity–related technology
Theme 3: technology usage habits and preferences	
3.1 Technology usage habits	Users’ general behaviors and routines related to technology usage
3.2 Preferred delivery modes	The preferences for certain types of sleep- and physical activity–related technologies

### Theme 1: Attitudes Toward the Use of Technology-Based Tools for Promoting Sleep and Physical Activity

#### Subtheme 1-1: Acceptance of New Technology-Based Tools/Strategies

Most participants (13/16, 81.3%) expressed willingness to try new strategies to address their sleep problems. When asked whether they were interested in SMS text messaging–based mobile health interventions for sleep, such as chatbots, most participants expressed a positive attitude. One participant mentioned that she had tried technology-based tools to improve sleep before without success, but she remained willing to try something new:

*I would be interested. […]. And I think if the chatbot also had some aspect of meditation. I think that that would be really useful too. I would love to try those. Those aren't things that I've tried to have helped. But those are things that I would love to try.*

[Participant 106, age 57, Focus Group 2]

In terms of physical activity, most participants expressed a positive attitude. In focus group 1, the interviewers and the participants discussed the willingness to participate in online exercise classes, and all participants demonstrated a positive willingness. The interviewer invited participants to reflect on the chat messages. One participant noted that the messages could provide motivation by serving as reminders to engage in exercise.

#### Subtheme 1-2: Concern About Technology Interfering With Sleep

Although most believed that developing such a tool had potential, one participant expressed concerns about the effectiveness of using phones and electronics to improve sleep.

*Checking phones and electronics right before bed probably doesn't help sleep or relaxation. I don't know*.

[Participant 113, age 52, Focus Group 3]

#### Subtheme 1-3: Data Privacy

When utilizing messaging-based mobile health interventions, users may be required to input their sleep and health conditions. Therefore, concerning data privacy, the research staff asked participants about their comfort with discussing personal health information over messaging apps or SMS text messaging. Some participants (4/16, 25%) agreed to share their personal experiences and conditions, while the rest did not express any opinion. None of them expressed opposition. Two participants answered:

*It’s fine. Like I said, I mentor people who are about to go through the same thing, so I'm able to help them with it. This part is coming at this stage of the chemo, and this is coming at that. And here’s, what’s going to happen at radiation so that they aren't blindsided.*

[Participant 202, age 41, Individual Interview 2]

*I like sharing similar situations. I think it’s helpful for me to talk about it. I think it’s helpful for me to hear other people’s similarities or things that are is they're getting help with.*

[110, Focus Group 3]

One focus group participant stated that the decision to share data relies on who is keeping it and the reason for keeping it, but generally, they would not oppose sharing it.

*I guess it would depend on who’s keeping it and why. I normally don't have a problem sharing.*

[Participant 113, age 52, Focus Group 3]

### Theme 2: Specific User Needs for Future Text Messaging–Based Interventions

#### Subtheme 2-1: Features

The focus groups and individual interviews discussed the needs and features that participants thought should be included in the development of a technology-based tool related to sleep and physical activity. Among these, reminder features were the most frequently mentioned (5/16, 31.3%). This feature could be used to remind users of their bedtime routine or meditation.

*If there was a bedtime routine, there’s something a sleep routine that would help with my sleep patterns, that would be great*.

[Participant 102, age 65, Focus Group 1]

*I think maybe a reminder now it’s time to put down your device and do some relaxing activity or meditation or relaxation exercise.*

[Participant 103, age 69, Focus Group 1]

*I use reminders at work all the time because I have so many things that I have to can't forget to do. And sometimes when you get busy you don't stop to make sure. So, I need reminders. So, to me, this would be very useful.*

[Participant 104, age 62, Focus Group 1]

Meditation or relaxation exercises were mentioned as commonly used strategies for improving sleep. Therefore, several participants (3/16, 18.8%) expressed a desire for the tool to include features that assist them in performing meditation or relaxation exercises.

*Maybe even suggest a — I don't know if that’s possible but — just some type of something you could do to help it, like a breathing exercise or giving you a place to go to find something that can [help] you get to sleep.*

[Participant 101, age 58, Focus Group 1]

*Any kind of activity for relaxation prior to bedtime would be really helpful*.

[Participant 102, age 65, Focus Group 1]

The capacity to analyze data for tracking and monitoring progress was one of the most frequently mentioned design features. Several participants (3/16, 18.8%) wanted to track their sleep progress and assess any improvements in their sleep quality.

*I really like that. Especially if you want to see — I like seeing my own improvement or the difference, you know, month over month, week over week*.

[Participant 201, age 47, Individual Interview 1]

In focus group 3, some participants mentioned the desire for social features to connect with others in similar situations and to support each other.

*I like sharing similar situations; I think it’s helpful for me to talk about them. I think it’s helpful for me to hear other people’s similarities or things that they're getting help with*.

[Participant 110, age 59, Focus Group 3]

However, one participant mentioned that she prefers to be on her own and does not need such a feature.

*I'm much more of a go it alone person, so that wouldn't be a thing for me.*

[Participant 109, age 60, Focus Group 3]

Two participants mentioned that they preferred having human experts available to provide guidance and interaction.

*I think that would be really important, especially if you know you're sharing, because your answer may not be cookie-cutter. So, to know that someone can decipher the nuances would be really good*.

[Participant 201, age 47, Individual Interview 1]

#### Subtheme 2-2: Time and Frequency

Given that reminders are among the most proposed features for tools designed to improve sleep, research staff further investigated how often users would like to be reminded and what would be the most appropriate times for these reminders. Summarizing their opinions, daily reminders and an hour before bedtime were deemed appropriate.

*I think daily would be fine*.

[Participant 103, age 69, Focus Group 1]

*An hour before bedtime or something like that. If they were talking about sleep-related, right? Maybe configurable, maybe start out every night and [see] if it’s too much.*

[Participant 113, age 52, Focus Group 3]

However, one participant indicated that receiving related information daily would make her feel stressed. She stated:

*No, I don’t want it every day. That would get on my nerves. I can't stand people like that*.

[Participant 203, age 56, Individual Interview 03]

#### Subtheme 2-3: Motivation for Engagement

For some participants, some features, such as tracking, reminders, sign-up, and goal setting, could help motivate their engagement with technology aimed at improving sleep and related behaviors such as physical activity. One participant shared her experience with a wearable device as an example, noting that the ongoing comparison of her progress serves as motivation for improvement.

*My son got me a Fitbit, one that would track, you know, steps and sleep. It was fascinating. I love it. It was motivating for me to see that every day, […] I like that, looking back, I just see I'm kind of competitive with myself. It’s like a contest. Like, oh, how well did I sleep, or what motivated me to do better? So tracking sticks out to me […]*.

[Participant 105, age 55, Focus Group 2]

In individual interview 1, participant 201 identified personal motivation as the primary barrier to sustaining regular physical activity. To address this, participant 201 endorsed accountability mechanisms and progress feedback. Some participants expressed that assistance with setting goals around sleep and physical activity would also be advantageous. One participant said:

*[…] It is important, at least for me, to have goals and to stick with them.*

[Participant 107, age 70, Focus Group 2]

Another participant believed that assistance with establishing and maintaining daily routines, as well as providing motivation during periods of inactivity, would be helpful for sustaining regular lifestyle habits.

*Maybe some short meditation too. Those are things that are hard to motivate yourself to do. But if it were a reminder, or it was on Zoom, and you could sign up for it, I think I would definitely do it*.

[Participant 103, age 69, Focus Group 1]

*If there’s somebody there to help you work through that and what it is, I think that would be helpful. [Both] at that moment, and then also motivate you if you haven't been working out, say for a few days. Get you to think about, maybe I need to go do some exercise. And I think that would be helpful*.

[Participant 103, age 69, Focus Group 1]

### Theme 3: Technology Usage Habits and Preferences

#### Subtheme 3-1: Technology Usage Habits

The research staff asked participants about their phone usage habits, specifically focusing on SMS text messaging. The majority of participants said that they frequently use this feature.

*Text messaging is one of the very few things I can do*.

[Participant 111, age 72, Focus Group 3]

*For everything, like I [do] texting, I still do work emails on it.*

[Participant 201, age 47, Individual Interview 1]

One participant said that they would use SMS text messaging but would encounter some minor limitations. When the researchers asked participants if they had experienced challenges with sending or receiving SMS text messaging, one participant responded:

*I do. We have a second home in a very remote part of Canada, so that becomes a challenge. But otherwise, bring it on!*

[112, Focus Group 3]

Two participants stated that they use this feature less frequently or that they use their phones less often in certain situations.

*I don't carry my phone with me when I'm working. It'll be in my office. So, um, I don't always have it on my person*.

[Participant 110, age 59, Focus Group 3]

*The reason I don't do texting is there are people on my phone who don't do texting. But in the past I loved to text because I don't have time to call.*

[Participant 203, age 56, Individual Interview 3]

Some individuals mentioned the utilization of technology-based tools such as apps and wearable devices to facilitate the implementation of these strategies. As noted in Participant 105’s quote in Subtheme 2‐3, using a Fitbit that tracked steps and sleep was described as fascinating and motivating.

*The nice thing about Minute is that it’s an app on my phone, so I can have it anywhere we are in the world and have my meditation if I have the issues. It works out really good*.

[Participant 107, age 70, Focus Group 2]

While many participants reported ownership of wearable devices and expressed a certain level of acceptance, one individual indicated that she could not use them due to allergies to nickel and plastic.

#### Subtheme 3-2: Preferred Delivery Modes

The focus group discussions included a conversation about the preferred type of technology-based intervention, such as SMS text messaging or an app. Four participants preferred using SMS text messaging, 4 participants preferred an app, and 3 were open to all of them. Two participants were neutral or uninterested in using SMS text messaging but did not oppose it.

*I like text. I think it’s easy, and you don't have to open up your email on your phone if you don't want to*.

[Participant 101, age 58, Focus Group 1]

*Text is best for me. And just to be able to access those relaxation techniques whenever I wanted them or needed them*.

[Participant 102, age 65, Focus Group 1]

*I think the app would be good because on that app, then you could have little things like relaxation tips or meditation, or you could have little things like that that people could then hit and follow*.

[Participant 103, age 69, Focus Group 1]

*I'd be open to all of them, but my number one preference would be the app*.

[Participant 106, age 57, Focus Group 2]

## Discussion

### Principal Findings

In our focus groups and individual interviews, we explored BC survivors’ perspectives on various approaches to delivering SMS text messaging–based mobile health interventions targeting sleep and physical activity. This included ascertaining the intervention’s format and potential functionalities. Three areas were focused upon in this study: attitudes toward SMS text messaging–based mobile interventions, technology preferences, and specific user needs.

To address sleep disturbances and improve physical activities, participants stated the need for an efficient intervention and expressed a positive attitude toward trying new approaches. Some participants mentioned that they were still seeking effective strategies. These findings highlighted the significance that participants attributed to sleep health and emphasized the need for suitable sleep-related interventions. Most participants exhibited a high level of acceptance toward technology, such as SMS text messaging and mobile apps, and they incorporated it into their daily lives. Prior studies showed that smartphone ownership among older adults increased significantly from 18% in 2013 to 83% for individuals aged 50 to 64 years and 61% for those aged 65 years and older in 2021 [33]. A survey from the American Association of Retired Persons in 2023 revealed that 94% of adults aged 50 years and above in the United States use SMS text messaging to maintain social connections [34]. This finding aligns with the usage habits reported by most of our interview participants. In combination with commonly used devices (eg, smartphones) and communication habits, this suggests that designing conversational interventions, such as interactive SMS text messaging or chatbots, is potentially feasible within this population.

In terms of features, incorporating a reminder related to sleep and physical activity was mentioned most frequently. A systematic review reported that personalized push reminders can positively influence users’ adherence [35]. Participants proposed that incorporating bedtime and relaxation exercise reminders could be helpful and that daily reminders, or notifications an hour before bed, would be acceptable. A systematic review paper regarding electronic health interventions in patients with BC indicates that users exhibited the most engagement with supportive features, including SMS text messaging, chat functions, and health reminders [36]. On the other hand, a significant decline in user engagement over time is also a common phenomenon [36]. A study with older patients with cancer suggested that customization features, such as the ability to ignore reminders, which give users more control, might enhance adherence to digital health interventions [37]. Consequently, when developing reminder features, user control and customization options should be considered fundamental components. The desire expressed by participants for mindfulness meditation tools aligns with literature, showing that mindfulness is effective for enhancing sleep quality among adults. Previous studies suggested that mindfulness meditation significantly improves sleep quality and reduces sleep-related daytime impairment [38,39]. Mindfulness meditation is thought to regulate arousal and neurocognitive processes, thereby mediating the link between how stimuli are perceived and how they are appraised [39]. Furthermore, a study implemented a meditation app to improve sleep in adults with sleep disturbances, resulting in significant reductions in presleep arousal and improvements in depression and anxiety [40]. Additionally, a qualitative study demonstrated that a mobile meditation tool to improve sleep quality is acceptable and feasible among adults, with tailoring needs varying by race and ethnicity [41].

Tracking features were also discussed in focus group interviews. The tracking feature can assist users in adhering to their goals, evaluating their progress, and motivating them to maintain healthy habits. In previous studies on technology-based interventions related to physical activity for the population with BC, tracking features have often been requested or incorporated [42,43]. Although there is a lack of studies specifically on the tracking features of sleep-related interventions for the population with BC, evidence from other clinical populations suggests potential benefits. A study with multiple sclerosis patients found that self-tracking tools helped clarify patterns related to symptoms, physical activity, and sleep quality and had a motivational effect on changing behavior [44]. Another research study focused on the general population indicated that routine self-tracking is more likely to provide positive modifications in health management strategies compared to event-triggered tracking [45]. In addition, the mindsets of users may influence the evaluation of self-tracking performance and affect the dynamics of the relationship formed with the tool [46]. If future studies incorporate self-tracking features into sleep-related interventions, researchers could evaluate their potential to enhance users’ motivation for lifestyle modifications and subsequently improve sleep quality.

In terms of technology preferences, both SMS text messaging and applications were mentioned as acceptable choices, with a shared consensus that mobile phones are the most convenient tool. According to prior studies, several sleep-related applications have been developed, some of which incorporate CBTi principles. These applications include features such as personalized sleep feedback, a sleep diary, and reminders [47]. However, there has been a lack of interventions delivered via SMS text messaging with interactive features, especially in the cancer survivors’ population. Participants who preferred SMS text messaging found texts to be easy to read and simple to operate, suggesting that this format holds potential as an effective intervention.

One such potential approach involves a conversational agent, including chatbots or other conversational interfaces, which mimic human conversation through spoken language or SMS text messaging [48]. Conversational agents are increasingly utilized in health care [49], and recent advances in large language models have also enabled an increasing use of conversational agents to support personal sleep wellness, for instance, by delivering CBTi–based interventions [50]. A customized SMS text messaging–based conversational agent tailored to this population could provide targeted support in real-time, delivering reminders, relaxation exercises, or sleep hygiene tips that are particularly relevant to their lived experiences. A growing body of research has emphasized the potential of SMS text messaging–based conversational agents in clinical practice and psychotherapy [51,52]. However, the application of such technology in addressing sleep and physical activity among BC survivors remains limited. We also asked participants about their perspectives on these developing technologies. Some participants expressed interest in using novel technologies such as SMS text messaging–based conversational agents for health interventions. Although novel technologies were regarded as acceptable in this study, one recent article discussed potential risks associated with chatbots. For instance, chatbots utilizing generative artificial intelligence may generate responses that cause psychological harm, provide misleading or inappropriate information, or lead the users overrely on them [53]. Therefore, these potential risks should be considered while developing the tools.

One participant expressed concerns regarding the use of electronic devices before bed. Prior research has shown that using smartphones in bed may negatively impact sleep quality, especially games that overload certain memory and cognitive functions. Conversely, smartphones used outside of bed do not decrease sleep quality [54]. Another research study also highlighted the complexity of the relationship between sleep quality and smartphone use. While screen use before bed may negatively affect sleep, if digital tools are used appropriately, some features, such as reminders, tailored recommendations, or other CBTi-related features, may still support users’ sleep and improve engagement [55]. Overall, this aspect should be considered when designing sleep-related digital tools to avoid any potential negative impact of technology on sleep.

### Future Directions

The long-term goal of this research is to help BC survivors improve their sleep and overall health. Accomplishing this goal will require a 2-fold effort: first, to develop an effective SMS text messaging–based mobile health intervention with strong adherence, such as a conversational agent, and second, to make this intervention widely available to the over 1 million BC survivors with insomnia living in the United States. The perspectives of BC survivors will be essential to each of the steps to determine where, when, and how an SMS text messaging–based mobile health intervention would be most useful as well as how to anticipate and address barriers and facilitators to access. To support the implementation and scalability of this intervention, we will also need to engage with BC clinicians to develop strategies to integrate the intervention into clinical practice.

In line with current technological trends, future interventions may leverage emerging technologies, such as conversational generative artificial intelligence and large language models, which have the potential to deliver personalized support tailored to BC survivors’ needs. Future research is needed to develop and evaluate the feasibility, acceptability, and effectiveness of this type of tool.

### Strengths and Limitations

One limitation of this study is that the demographic composition of the sample mainly comprised older adults with higher levels of education, and the majority of participants self-identified as White. In addition, most participants were based in the United States, with recruitment primarily targeted in Washington State. The lack of a diverse population included in this study may limit the generalizability of the findings to broader populations. Moreover, this study used social media for recruitment, and the interviews were conducted virtually via Zoom. As a result, most of the participants were likely to have a greater degree of knowledge and familiarity with using technology. The perspectives of groups less familiar with using technology were not included. However, the findings could still provide meaningful insights into the specific group perspectives and serve as a foundation for developing SMS text messaging–based mobile health interventions to promote sleep and physical activity. We also recommend that future research be expanded to include more diverse populations to enhance the breadth of perspectives represented in the findings.

### Conclusions

We gathered perspectives from BC survivors to inform the design of an SMS text messaging–based mobile health intervention, which incorporated common sleep-related interventions such as CBTi and physical activity. The participants generally expressed openness to SMS text messaging–based mobile interventions and potential features such as tracking and reminders. The preference for delivery modes such as SMS text messaging could serve as a reference for tailoring interventions to this population.

## Abbreviations

BC: breast cancer
CBTi: cognitive behavioral therapy for insomnia
